# Autonomic nervous system dysregulation in response to postural change in patients with pectus excavatum in Taiwan: a pilot study

**DOI:** 10.1186/s13019-022-01835-5

**Published:** 2022-05-03

**Authors:** Yu-Ting Hsu, Yeung-Leung Cheng, Yi-Wei Chang, Chou-Chin Lan, Yao-Kuang Wu, Mei-Chen Yang

**Affiliations:** 1 Physical Therapist, Department of Rehabilitation Medicine, Taipei Tzu Chi Hospital, Buddhist Tzu Chi Medical Foundation, New Taipei, Taiwan; 2 Division of Thoracic Surgery, Department of Surgery, Taipei Tzu Chi Hospital, Buddhist Tzu Chi Medical Foundation, New Taipei, Taiwan; 3 School of Medicine, Tzu Chi University, Hualien, Taiwan; 4 Department of Rehabilitation Medicine, Taipei Tzu Chi Hospital, Buddhist Tzu Chi Medical Foundation, New Taipei, Taiwan; 5 Division of Pulmonary Medicine, Department of Internal Medicine, Taipei Tzu Chi Hospital, Buddhist Tzu Chi Medical Foundation, No. 289, Jianguo Rd., Xindian Dist., New Taipei City, 23143 Taiwan

**Keywords:** Autonomic nervous system, Pectus excavatum, Postural change, Nuss surgery

## Abstract

**Background:**

Pectus excavatum (PE) negatively impacts psychological function, but its effect on autonomic nervous system (ANS) function has not been investigated. We evaluated ANS function following postural changes in patients with PE.

**Methods:**

The participants were 14 healthy men (control group) and 20 men with PE (study group). Psychological function was assessed using the visual analog scale for pain, Brief Symptom Rating Scale-5, and Beck Depression Inventory-II. Sleep quality was evaluated using the Pittsburgh Sleep Quality Index (PSQI). ANS regulation in response to postural change was measured in the supine position and immediately after sitting. All measurements were compared between the control and study groups at baseline and between the study groups before and after Nuss surgery.

**Results:**

At baseline, upon postural change, symptomatic activity increased in the control group (50.3–67.4%, p = 0.035) but not in the study group (55.0–54.9%, p = 0.654); parasympathetic activity decreased in the control group (49.7–32.6%, p = 0.035) but not in the study group (45.1–45.1%, p = 0.654); and overall ANS regulation increased in the control group (1.02–2.08, p = 0.030) but not in the study group (1.22–1.22, p = 0.322). In response to postural change after Nuss surgery in the study group, sympathetic activity increased (48.7–70.2%, p = 0.005), parasympathetic activity decreased (51.3–29.8%, p = 0.005), and overall ANS regulation increased (0.95–2.36, p = 0.012).

**Conclusion:**

ANS function in response to postural change is dysregulated in patients with PE, which improved after Nuss surgery.

*Trial registration* ClinicalTrials.gov, ID: NCT03346876, November 15, 2017, retrospectively registered, https://register.clinicaltrials.gov/prs/app/action/SelectProtocol?sid=S0007KGI&selectaction=Edit&uid=U0003JZU&ts=2&cx=cstxeg

## Background

The autonomic nervous system (ANS) is an important homeostatic control system that regulates bodily functions, such as heart rate, blood pressure, and respiratory rate. Furthermore, it is the primary mechanism of controlling the fight-or-flight response to adapt to environmental changes [1]. Generally, the ANS has two branches, the sympathetic and the parasympathetic, which act reciprocally [2], and respond to the circadian rhythm of the body [3]. The sympathetic nervous system is often considered the “fight or flight” system, while the parasympathetic nervous system is considered the “rest and digest” system. Sympathetic activity is reported to increase in some normal physiological conditions (tilting, standing, mental stress, moderate exercise) and in certain pathological conditions (hypotension, coronary artery occlusion, cerebrovascular accident) [1]. Parasympathetic activity is increased during controlled respiration, cold stimulation, or rotational stimuli [1].

Heart rate variability (HRV) is one of the most promising standard measurements and provides valuable insights into the associations between physiological/pathological conditions and many diseases, particularly acute myocardial infarction, hypertension, arrhythmia, and psychiatric disorders [1, 4, 5].

Pectus excavatum (PE) has been found to be associated with physiological and psychological impairments [6, 7]. While ANS function might be affected in patients with PE, it has never been evaluated. Therefore, this study aimed to evaluate ANS function in response to postural changes in patients with PE.

## Methods

### Participants

A total of 93 patients with PE scheduled for the Nuss surgery were invited to participate between August 2017 to July 2018. The inclusion criteria were as follows: age 13–45 years, non-employed, Haller index [8] ≥ 3.0, no known psychiatric or medical illness, and no use of psychoactive, soporific, or illegal drugs. The exclusion criteria were as follows: age < 13 or > 45 years; Haller index < 3.0; having major medical diseases, known psychiatric disease, or known sleep-disordered breathing, combined with other musculoskeletal diseases, and having undergone another major surgery within the past 6 months. After excluding patients who did not complete questionnaires, had an error while obtaining HRV measurements, and refused to participate in the study, a total of 43 participants were included. After Nuss surgery, 19 of 43 patients were followed up at another medical center, and 4 of 43 patients were lost to follow-up due to the study being conducted overseas. The final 20 patients completed the same measurements 6 months after the Nuss surgery.

Additionally, another 15 non-employed, healthy participants were eligible for comparison and recruited from the families of patients with PE or from among our medical staff and their families; they fulfilled the same inclusion criteria but had no PE. All healthy participants, except for one, completed all measurements at baseline. For the final analysis, 14 healthy participants were included in the control group.

### Anthropometric measurements and demographic data

Baseline clinical characteristics, including age, sex, body height, body weight (BW), body mass index (BMI), and smoking status were recorded.

### Physiological and psychological function measurements

The evaluation questionnaires included the visual analog scale for pain (VAS), the Chinese version of the five-item Brief Symptom Rating Scale (BSRS-5) [9], the validated Chinese version of the Beck Depression Inventory-II (BDI-II) [10], and the validated Chinese version of the Pittsburgh Sleep Quality Index (PSQI) questionnaires [11].

The VAS rated the severity of painful bodily sensations, ranging from 0 (no pain) to 10 (extremely severe pain).The BSRS-5 is a five-item questionnaire that measures anxiety (tense or high-strung), depression (depressed or in a low mood), hostility (easily annoyed or irritated), interpersonal sensitivity (feeling inferior to other people), and additional symptoms (having trouble falling asleep in the past 1 week). Each item score ranges from 0 to 4 (0 = not at all; 1, a little bit, 2 = moderately; 3, quite a bit; 4, extremely).

The BDI-II® questionnaire measures the severity of subjective depression in adolescents and adults. It contains 21 items, and each item is scored from 0 to 3 (0 = do not feel so; 1 = feel so; 2 = feel so all the time and cannot snap out of it; 3, feel so that cannot stand it). The sum of the 21 items (BDI-II score) indicates the severity of depressive symptoms (higher BDI-II score, more severe depressive symptoms).

The PSQI questionnaire assesses subjective sleep quality, containing seven components: sleep quality, sleep latency, sleep duration, sleep efficiency, sleep disturbance, frequency of sleeping medication use, and daytime functional impairment. Each PSQI component was rated from 0 to 3 (0 = no difficulty; 3 = extreme difficulty). The sum of these seven components (PSQI score) represents the global subjective sleep quality (good and poor sleep quality corresponding to a PSQI score ≤ 5 and ≥ 6, respectively).

### Evaluation of ANS regulation in response to postural change

HRV was evaluated using the 5-min short-term recording by the HRV analysis software [CheckMyHeart (CMH), Taiwan] Version 3.0. The CMH system included a single-lead electrocardiography (ECG) recorder (lead I or lead II). Beat-by-beat RR interval values (resolution 4 ms) were obtained from the ECG signals from the CMH software, which automatically rejected and allowed manual filtering of irregular RR intervals. The detrended time series were cubically interpolated and resampled at 1 Hz. After detrending via least-squares second-order polynomial fitting, the power spectral density of the RR interval time series was estimated by discrete Fourier transform, which was performed using an autoregressive method (Burg algorithm) with spectral decomposition (Johnsen and Andersen algorithm). Powers in the very-low-frequency (VLF, 0.00–0.04 Hz), low-frequency (LF, 0.04–0.15 Hz), and high-frequency (HF, 0.15–0.40 Hz) bands were obtained by numerical integration. Components showing < 10% of the overall power in the band were ignored, as they might represent noise contributions. The spectral powers of the VLF, LF, and HF bands were computed as the sum of the respective spectral components. Frequency domain methods were used, and the LF and HF were expressed in normalized units (n.u.) to minimize the effect on the values of LF and HF components of the changes in total power, calculated as follows: (absolute power of the components)/(total − VLF power) × 100. The physiological correlations of VLF are still unclear; therefore, we did not collect VLF values. As the short-term HRV measurements rapidly returned to baseline after transient or mild perturbations, such as milder activity and postural change, we obtained HRV measurements while the participants were in the supine posture and immediately upon sitting up. The LFn.u., HFn.u., and LF/HF ratios were recorded in these two postures. Usually, the LFn.u. represents sympathetic activity, and the HFn.u. represents parasympathetic activity; the LF/HF ratio indicates the overall ANS regulation [1].

### Statistical methods

All continuous variables were non-normally distributed and presented as medians and interquartile ranges (Q1, Q3). Categorical variables were shown as numbers and percentages (%). For comparison between the study and control groups at baseline, the Mann–Whitney U test was used for analyzing continuous variables. The Pearson's chi-square was used to analyze categorical variables.. For comparison before and after Nuss surgery among the study group, the Wilcoxon signed ranks test was used to analyze continuous variables, and the McNemar’s test was used to analyze categorical variables. All statistical assessments were two-tailed and considered significant at p < 0.05. All statistical analyses were performed using IBM SPSS statistical software version 24 for Windows (IBM Corp., Armonk, New York, USA).

## Results

At baseline, there were no between group differences in age, sex, smoking status, BSRS-5, BDI-II, PSQI, and ANS function. The patients with PE weighed lesser than the control group (median BW: 57.4 kg vs. 65.2 kg, p = 0.003; median BMI: 18.5 kg/m^2^ vs. 21.5 kg/m^2^, p = 0.001) and reported greater painful bodily sensation (median VAS scale 0 [IQR 0, 2.3] vs. 0 [IQR 0, 0], p = 0.008) (Table 1).

**Table 1:** Comparison of demographics, quality of life, and autonomic nervous regulation between the control and study groups among male non-employed participants at baseline

	Control	Study	p-value
	(n = 14)	(n = 20)	
Haller index	N/A	3.6(3.4, 4.0)	N/A
Basic information			
Age, years old	21.0 (19.8, 23.3)	19.0 (16.0, 22.0)	0.144
Smoking status			1.000
Non-smoker, n (%)	14 (100.0%)	19 (95.0%)	
Smoker, n(%)	0 (0.0%)	1 (5.0%)	
Body height, cm	175.0 (170.9, 181.3)	174.3 (169.2, 176.8)	0.517
Body weight, kg	65.2 (59.3, 69.6)	57.4 (48.3, 61.8)	0.003*
Body mass index, kg/m^2^	21.5 (19.6, 22.4)	18.5 (16.9, 20.6)	0.001*
Psychological functions			
BSRS-5	4.0 (1.5, 7.0)	2.0 (0.3, 7.0)	0.446
VAS	0 (0,0)	0 (0, 2.3)	0.008*
BDI-II	1.0 (0, 4.3)	1.0 (0, 7.0)	0.928
PSQI score	4.0 (2.0, 7.3)	4.0 (2.3, 6.0)	0.929
PSQI sleep quality			1.000
Poor, PSQI≧6, n (%)	5 (36.0%)	7 (35.0%)	
Good, PSQI≦5, n (%)	9 (64.0%)	13 (65.0%)	
ANS activity (supine posture)			
LF nu,%	50.3 (36.9,60.4)	55.0 (45.3,67.8)	0.506
HF nu,%	49.7 (39.6,63.1)	45.1 (32.2,54.7)	0.484
LF/HF	1.02 (0.59,1.54)	1.22 (0.83,2.11)	0.506

Continuous variables were summarized as median (IQR) and were analyzed using the Mann–Whitney U test. Categorical variables were summarized as n (%) by group and were analyzed using Fisher's Exact test. All statistical assessments were two-tailed and considered significant as p < 0.05. Bold font highlight the statistic significance with p < 0.05

BDI-II, Beck Depression Inventory version II; BSRS-5, Brief Symptom Rating Scale-5; IQR, interquartile range; PSQI, Pittsburgh Sleep Quality Index; VAS, visual analog scale for pain

**Fig. 1:**
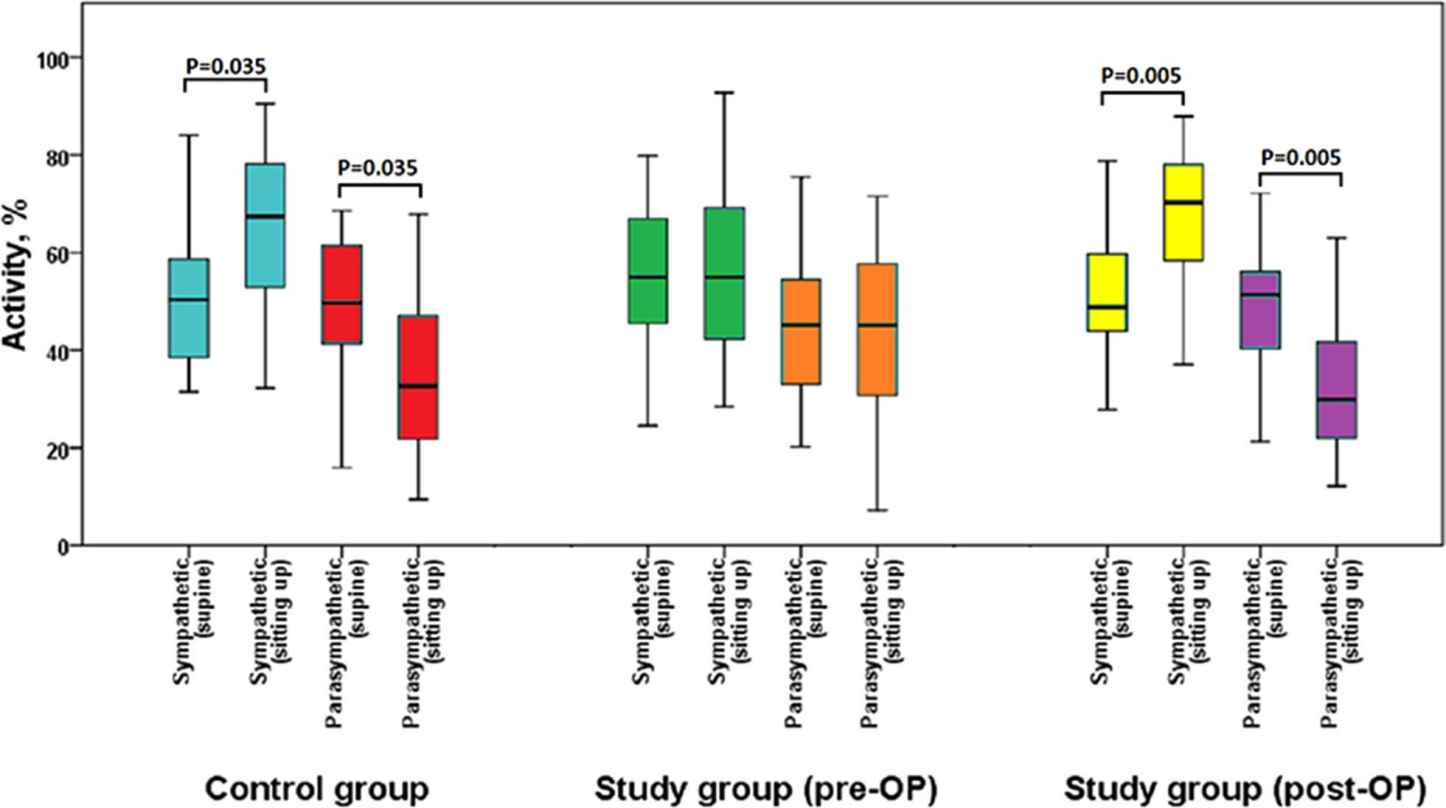
The autonomic nervous system function in response to postural change in the control group at baseline and in the study group before and after Nuss surgery

Upon postural change, the sympathetic activity significantly increased in the control group (from 50.3 to 67.4%, p = 0.035), but no change was observed in the study group (from 55.0 to 54.9%, p = 0.654); moreover, the parasympathetic activity significantly decreased in the control group (from 49.7 to 32.6%; p = 0.035), but no change was observed in the study group (from 45.1 to 45.1%, p = 0.654) (Table 2 and Fig. 1). The overall ANS regulation significantly increased in response to postural change in the control group (from 1.02 to 2.08, p = 0.030), but not in the study group (from 1.22 to 1.22, p = 0.322) (Table 2).

**Table 2:** Autonomic nervous regulation in responsible to postural change of the control group and study groups at baseline and after operation among male non-employed participants

	Control (n = 14) at baseline			Study (n = 20) at baseline			Study (n = 20) after operation		
	Supine	Sitting up	p-value	Supine	Sitting up	p-value	Supine	Sitting up	p-value
LF nu,%	50.3 (36.9,60.4)	67.4 (52.5,79.8)	0.035*	55.0 (45.3,67.8)	54.9 (41.4,71.5)	0.654	48.7 (43.9,60.2)	70.2 (56.0,78.5)	0.005*
HF nu,%	49.7 (39.6,63.1)	32.6 (20.2,47.5)	0.035*	45.1 (32.2,54.7)	45.1 (28.5,58.6)	0.654	51.3 (39.8,56.1)	29.8 (21.5,44.0)	0.005*
LF/HF	1.02 (0.59,1.54)	2.08 (1.11,4.05)	0.030*	1.22 (0.83,2.11)	1.22 (0.71,2.58)	0.322	0.95 (0.78,1.51)	2.36 (1.29,3.67)	0.012*

Continuous variables were presented as median (IQR) and were analyzed using the Wilcoxon Signed Ranks test. All statistical assessments were two-tailed and considered significant as p < 0.05. Bold font highlight the statistic significance with p < 0.05

HF, high frequency; IQR, interquartile range; LF, low frequency; n.u., normalized units

After Nuss surgery, the study group had reported an increase in painful bodily sensation (median VAS 0 vs. 2, p = 0.032), but there were no changes in the BSRS-5, the BDI-II score, the PSQI score, and in the subjective PSQI sleep quality (Table 3). In response to postural change after Nuss surgery in the study group, the sympathetic activity increased (48.7–70.2%, p = 0.005); the parasympathetic activity decreased (from 51.3 to 29.8%, p = 0.005), and the overall ANS activity increased (from 0.95 to 2.36, p = 0.012) (Table 2 and Fig. 1).

**Table 3:** Comparison of quality of life before and after Nuss surgery among male non-employed patients

	Before Nuss surgery	After Nuss surgery	p-value
	n = 20	n = 20	
BSRS-5	2.0 (0.3, 7.0)	1.0 (0, 4.0)	0.149
VAS	0 (0, 2.3)	2.0 (1.0, 3.0)	0.032*
BDI-II	1.0 (0, 7.0)	2.0 (1.0, 3.8)	0.705
PSQI score	4.0 (2.3, 6.0)	4.5 (3.0, 6.8)	0.288
PSQI sleep quality			1.000
Poor, n (%) (PSQI ≥ 6)	7 (35.0%)	7 (35.0%)	
Good, n (%) (PSQI≦5)	13 (65.0%)	13 (65.0%)	

Continuous variables were presented as median (IQR), and were analyzed by the Wilcoxon Signed Ranks Test. Categorical variables were presented as count (%) and were analyzed by McNemar's Test

*All statistical assessments were two-tailed and considered significant as p < 0.05. Bold font highlight the statistic significance with p < 0.05

BDI-II: Beck Depression Inventory version 2; BSRS-5: Brief Symptom Rating Scale; IQR, interquartile range; PSQI: Pittsburgh Sleep Quality; VAS: Visual Analogue Scale for pain

## Discussion

We found that the bilevel ANS regulation in response to postural change was lower in patients with PE than in the general population, which improved after Nuss surgery.

Normally, upon postural change, sympathetic activity increases and parasympathetic activity decreases to maintain blood pressure and avoid the occurrence of postural hypotension [1]. Our study showed normal ANS regulation in response to postural changes among healthy participants. Therefore, the abnormal ANS regulation among patients with PE in our study might truly exist even though we only included a small number of cases. We did not measure the blood pressure to evaluate ANS dysregulation associated with postural hypotension while obtaining HRV records as this maneuver might affect the HRV results. Therefore, our results could not assess the relationship between ANS dysregulation and postural hypotension in patients with PE. Thus, further studies are needed to establish the absence or presence of postural hypotension and how ANS dysregulation and postural hypotension affect patients with PE.

Furthermore, many studies have found that short-term HRV measurements rapidly return to baseline after transient perturbations induced by some postural changes (such as mild exercise, milder activity, postural change, short-acting vasodilators, transient coronary occlusion, etc.) [1]. More powerful stimuli (such as maximum exercise or long-acting drugs) may result in much more prolonged HRV changes. We chose postural change as a measure to evaluate ANS function between healthy participants and patients with PE because of its high feasibility and low invasiveness. The effects of PE on ANS function in response to other minor and major stresses remain unknown and require further examination.

The painful bodily sensation was found to be associated with ANS dysfunction, with higher sympathetic and lower parasympathetic activity [12–14]. Bodily pain has been reported in patients with PE due to an asymmetrical chest wall associated with connective tissue abnormalities. In our study, we did not observe the trend of higher sympathetic and lower parasympathetic activities in patients with PE who had more bodily painful sensations than healthy participants before surgery. Postoperatively, there was an increase in the reported pain in patients with PE, but ANS regulation normalized. In a previous study, the authors found that ANS reactivity to pain could be altered by environmental and psychological factors throughout an individual’s life [15]. These results indicate that beyond the painful bodily sensation there might be additional factors, or the changes associated with PE per se, affecting ANS regulation in this population The relationship between pain and ANS dysregulation among patients with PE requires further evaluation.

### Sex differences in autonomic nervous system regulation

The sex differences in ANS regulation have been well documented previously in a healthy population; men were found to have more pronounced sympathetic influence than women [16, 17]. Daunt SW studied the chest wall configuration as measured by the Haller index in normal children and found that healthy women had a higher Haller index than men [18] and suggested that sex-related differences should be considered when evaluating children with chest wall deformity. PE has a higher prevalence in men. In our study, only male participants were enrolled, and this ANS dysregulation might be different among female participants, and further research is needed.

### Cigarette smoking affects autonomic nervous system regulation

Cigarette smoking has been found to increase sympathetic activity [19]. There were no smokers in our control group and only one smoker in our study group. Although the percentage of smoking status was not statistically different, due to our small sample size, the results of our study might be underestimated, and further research is needed.

### Unemployment status and the autonomic nervous system regulation

Jandackova et al. examined ANS function using a modified orthostatic test by measuring ANS function in three sequential postures (supine, standing, supine) in involuntary non-employed and employed participants aged 30–49 years, matched on sex, type of job, health-related behavior, and BMI. They found that involuntary non-employed participants had decreased parasympathetic activity and suggested that seeking a job might be a potential chronic stress leading to suppression of parasympathetic activity [20, 21]. In our study, only non-employed participants were enrolled. Our participants were younger and recent graduates from senior high schools or colleges and were deemed as voluntary non-employed because they scheduled the surgery before “starting to seek a job”. The economic stress might therefore, be different for our participants and might not have such an impact on ANS function. Even in employed individuals, work stress has an adverse effect on HRV regulation [22, 23]. Moreover, Maeda et al. found that home stress could induce ANS imbalance, even in employed people [24]. These results indicate that employment status was not the only confounding factor in HRV regulation.

### Influence of the autonomic nervous system on body weight control

Molfino et al. reported an involvement of the parasympathetic system in BW regulation; lower parasympathetic activity is observed with higher BW [25]. Another study by Koenig et al. an association of parasympathetic activity with increased energy consumption for bodily functions, resulting in decreased BW and BMI in non-obese healthy people [26]. Although our study group had lower BW and BMI, the control group participants were also non-obese, which might explain why the baseline ANS activity was not different between these two groups.

### Physiological and psychological impairments and autonomic nervous system regulation

Jiang et al. found a reduced sympathetic and accentuated parasympathetic response to postural changes in patients with depressive disorders [27]. Wang et al. found that depression severity is linked to the severity of ANS dysfunction [28]. In our previous study [29], we found that patients with PE had a more depressive mood. However, the participants were older and included both, employed and non-employed individuals and men and women. In the present study, we did not observe any difference in ANS dysfunction between healthy participants and patients with PE, and among patients with PE before and after surgery. This is possibly because our study participants were all male and non-employed. Thus, ANS dysfunction might differ according to sex, age, and employment status.

## Limitations

This study had several limitations. First, the sample size was small and might not be representative of all patients with PE. Second, all questionnaires to evaluate psychological impairments were self-reported, and therefore, were subjective. Although our participants received assistance to ensure questionnaire integrity, there were no psychological counselors or psychiatrists that validated the responses. Thus, a depressive mood may have an effect on the responses. Third, as all our participants were Taiwanese, there might be ethnic and cultural differences in physical and psychosocial conditions. Finally, we only found differences between the young and non-employed male participants. The real association between ANS function and pectus deformity needs further exploration, regardless of age, sex, and smoking status. The impact of PE severity on ANS function also needs further understanding.

## Conclusions

We found reduced ANS regulation in response to a postural change in male non-employed patients with PE, which improved after Nuss surgery. Moreover, there may be an association between ANS dysfunction and PE. Lastly, ANS function evaluation might be used as an auxiliary tool to evaluate the physiological and psychological impacts of PE.

## Data Availability

All data generated or analyzed during this study are included in this published article.

## Abbreviations

ANS: Autonomic nervous system
BMI: Body mass index
BW: Body weight
HF: High-frequency
HRV: Heart rate variability
LF: Low-frequency
PE: Pectus excavatum
PSQI: Pittsburgh Sleep Quality Index
VLF: Very-low-frequency
